# Case Report: Early-onset portal hypertension revealing pediatric ADPKD with a truncating PKD1 variant

**DOI:** 10.3389/fped.2026.1851979

**Published:** 2026-10-01

**Authors:** Ana Katerin Minota Idarraga, Ivan Mauricio Llanos Arango

**Affiliations:** 1 Universidad Libre Seccional Cali, Cali, Colombia; 2 GRINPED Pediatric Research Group, Cali, Colombia

**Keywords:** autosomal dominant, ciliopathies, congenital hepatic fibrosis, esophageal variceal bleeding, PKD1 truncating variant, portal hypertension

## Abstract

**Background:**

Autosomal dominant polycystic kidney disease (ADPKD) is a multisystemic ciliopathy whose clinical spectrum extends into childhood. Although hepatic cysts are the most common extrarenal manifestation, early portal hypertension associated with congenital hepatic fibrosis (CHF)-like features is uncommon and likely underrecognized.

**Case presentation:**

We report a 10-year-old girl with no known family history of ADPKD who presented at 9 years of age with upper gastrointestinal bleeding secondary to grade III esophageal varices. The diagnostic evaluation established non-cirrhotic portal hypertension, and she underwent endoscopic variceal ligation followed by secondary prophylaxis with propranolol. Imaging demonstrated hepatosplenomegaly, patent portal and splenic veins, and no hepatic cysts. Liver biopsy showed mild portal fibrosis (METAVIR F1), while liver stiffness measured 6.4 kPa, consistent with mild fibrosis. Renal imaging revealed multiple bilateral cortical cysts, and whole-exome sequencing identified a heterozygous truncating PKD1 variant (p.Glu1633Ter), classified as pathogenic according to ACMG criteria. The patient remains clinically stable without recurrent bleeding during multidisciplinary follow-up.

**Discussion:**

This case expands the phenotypic spectrum of pediatric ADPKD by illustrating clinically significant portal hypertension despite only mild hepatic fibrosis. It also highlights the importance of integrating histopathology, non-invasive liver assessment, imaging, and molecular testing when evaluating children with unexplained non-cirrhotic portal hypertension.

**Conclusion:**

ADPKD should be considered in the differential diagnosis of pediatric non-cirrhotic portal hypertension, even in the absence of hepatic cysts. Early genetic testing may facilitate accurate diagnosis and appropriate multidisciplinary management.

## Introduction

Autosomal dominant polycystic kidney disease (ADPKD) is one of the most common inherited kidney disorders, affecting approximately 1 in 400 to 1,000 individuals worldwide, and represents a major cause of chronic kidney disease and kidney failure (1–3). The disease is primarily caused by pathogenic variants in **PKD1** and **PKD2**, which encode polycystin-1 (PC1) and polycystin-2 (PC2), two ciliary proteins involved in mechanosensation, calcium signaling, epithelial polarity, and tubular morphogenesis (2, 3). Dysfunction of this complex results in progressive bilateral renal cyst formation and variable extrarenal manifestations.

Although traditionally regarded as an adult-onset disorder, ADPKD is increasingly recognized as a disease with a broad clinical spectrum that includes childhood presentations and, in some patients, very early-onset disease (VEO-ADPKD) (1, 2). Truncating **PKD1** variants have consistently been associated with more severe renal phenotypes, including larger kidney volumes and more rapid progression of renal dysfunction (4).

Beyond the kidneys, ADPKD is now recognized as a multisystemic ciliopathy. Polycystic liver disease is its most frequent extrarenal manifestation, whereas portal hypertension associated with congenital hepatic fibrosis (CHF)-like features represents an uncommon but increasingly recognized phenotype (5–8). CHF belongs to the spectrum of ductal plate malformations and is characterized by portal fibrosis, abnormal bile duct remodeling, and preservation of hepatocellular architecture (8, 9). Clinically, affected patients may develop splenomegaly, hypersplenism, esophageal varices, and upper gastrointestinal bleeding despite preserved liver synthetic function (7, 9).

Although CHF is classically associated with autosomal recessive polycystic kidney disease (ARPKD), several reports have described similar hepatic manifestations in patients with ADPKD, particularly in those carrying truncating **PKD1** variants (4, 6). These observations suggest that severe hepatorenal involvement may occur across different ciliopathies, although the underlying mechanisms remain incompletely understood (2, 3, 10).

In children, portal hypertension may occasionally precede clinically evident renal disease, making recognition of the underlying disorder particularly challenging (4, 6). The differential diagnosis of pediatric non-cirrhotic portal hypertension is broad and includes vascular, infectious, metabolic, hematologic, immunologic, and drug-related disorders in addition to congenital liver diseases. Increasingly, genetic testing has become an essential component of the diagnostic evaluation in children with unexplained portal hypertension, particularly when accompanied by features suggestive of a ciliopathy.

We report a child who presented with severe upper gastrointestinal bleeding secondary to non-cirrhotic portal hypertension as the initial manifestation of ADPKD associated with a pathogenic truncating **PKD1** variant, highlighting the importance of integrating clinical, radiologic, histopathologic, and molecular findings for accurate diagnosis.

## Case presentation

Figure 1 was created with the assistance of OpenAI's ChatGPT image generation tools. The authors conceived the figure, provided all clinical content, verified the scientific accuracy of the information, and reviewed and approved the final version.

**Figure 1:**
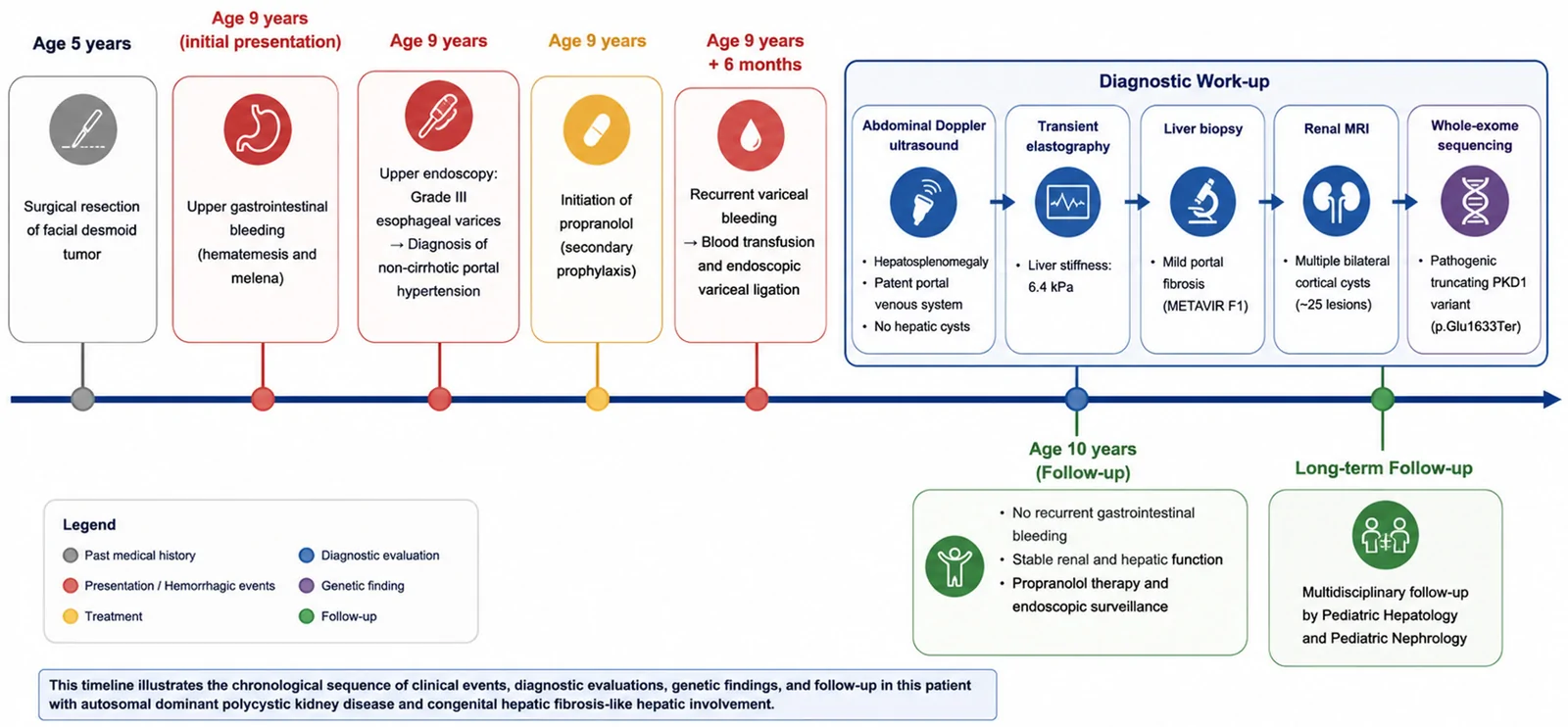
Clinical timeline of the patient.

The patient's chronological clinical course, from the initial presentation through diagnostic evaluation, therapeutic interventions, genetic diagnosis, and follow-up, is summarized in Figure 1. A 10-year-old girl was referred for evaluation after presenting at 9 years of age with upper gastrointestinal bleeding manifested by hematemesis and melena. Upper gastrointestinal endoscopy revealed grade III esophageal varices (Figure 2). Further evaluation demonstrated clinically significant non-cirrhotic portal hypertension, and secondary prophylaxis with propranolol was initiated. Approximately six months later, she experienced recurrent variceal bleeding requiring packed red blood cell transfusion and endoscopic variceal ligation (Figure 2). Laboratory findings obtained during this episode are summarized in Table 1.

**Figure 2:**
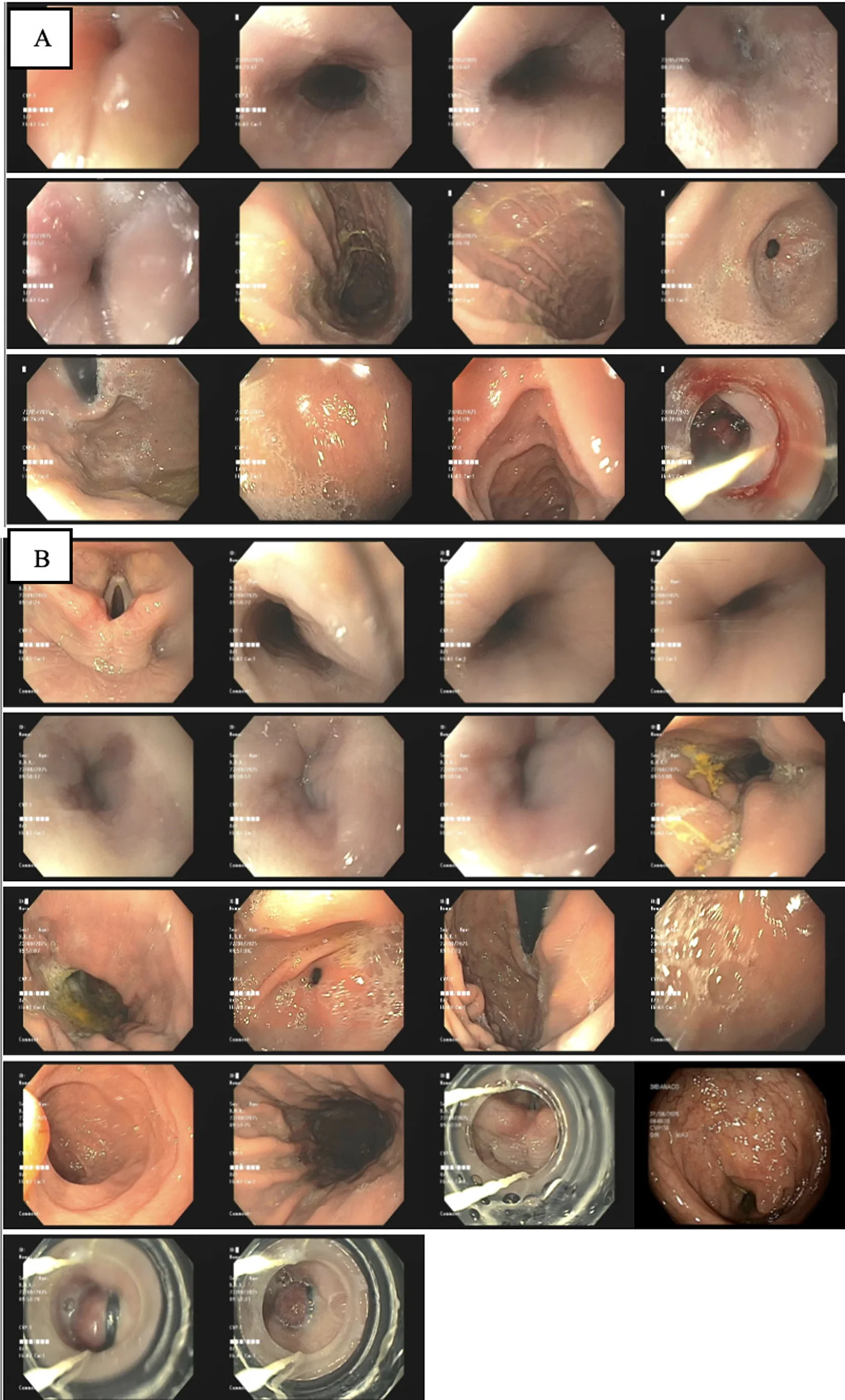
Upper gastrointestinal endoscopy at baseline and 3-month follow up. **(A)** initial upper gastrointestinal endoscopy performed during gastrohepatology follow-up demonstrated large esophageal varices (baveno classification) without active bleeding, consisting of a single variceal column. Endoscopic variceal ligation was performed with placement of one band. Mild portal hypertensive gastropathy was also observed. **(B)** Follow-up upper gastrointestinal endoscopy at 3 months confirmed persistent portal hypertension, with a large esophageal varix (single column, Baveno classification). Endoscopic variceal ligation was repeated with placement of one band. Mild portal hypertensive gastropathy persisted.

**Table 1:** Summary of clinical, laboratory, imaging, histopathologic, and genetic findings.

Category	Parameter	Result
Clinical presentation	Initial presentation	Hematemesis and melena
	Portal hypertension	Grade III esophageal varices
	Splenomegaly	Present
Hematology	Hemoglobin	5 g/dL
	Platelets	98 × 10^3^/µL
Liver biochemistry	AST	32 U/L
	ALT	21 U/L
	Albumin	4.87 g/dL
Imaging	Doppler ultrasound	Hepatosplenomegaly, patent portal venous system, no hepatic cysts
	Transient elastography	6.4 kPa
	Renal MRI	Multiple bilateral cortical cysts (∼25 cortical cysts)
Histopathology	Liver biopsy	Mild portal fibrosis (METAVIR F1), compatible with congenital hepatic fibrosis-like changes
Genetics	Whole-exome sequencing	PKD1 c.4897G > T (p.Glu1633Ter)
	ACMG classification	Pathogenic

She was the daughter of non-consanguineous parents and had been born at term (39 weeks of gestation) by cesarean delivery without perinatal complications. Birth weight was 3,600 g, and no neonatal hospitalization was required. Her past medical history was remarkable only for surgical resection of a facial desmoid tumor at 5 years of age. Growth and development had been normal. She had no previous history of liver disease, thrombotic events, or chronic systemic disorders. Family history was notable for nephrolithiasis in her mother and in a second-degree relative, whereas her father was healthy. There was no known family history of polycystic kidney disease, chronic liver disease, or inherited genetic disorders.

Given the recurrence of variceal bleeding in the absence of established cirrhosis, a comprehensive diagnostic evaluation was performed. Metabolic, autoimmune/immunologic, hematologic, infectious, and drug-related causes of liver disease and portal hypertension were systematically excluded through appropriate biochemical, serological, and metabolic investigations (Table 1).

Abdominal Doppler ultrasound demonstrated mild hepatomegaly with heterogeneous echogenicity and splenomegaly (Figure 3). The portal and splenic veins were patent, with preserved hepatopetal portal flow and normal visualization of the intrahepatic portal branches. No hepatic cysts, focal liver lesions, biliary duct dilatation, ascites, or portal venous thrombosis were identified. Liver stiffness measured by transient elastography was 6.4 kPa, consistent with mild fibrosis. Percutaneous liver biopsy demonstrated mild chronic hepatitis with minimal inflammatory activity and portal fibrosis (METAVIR F1) (Table 2).

**Figure 3:**
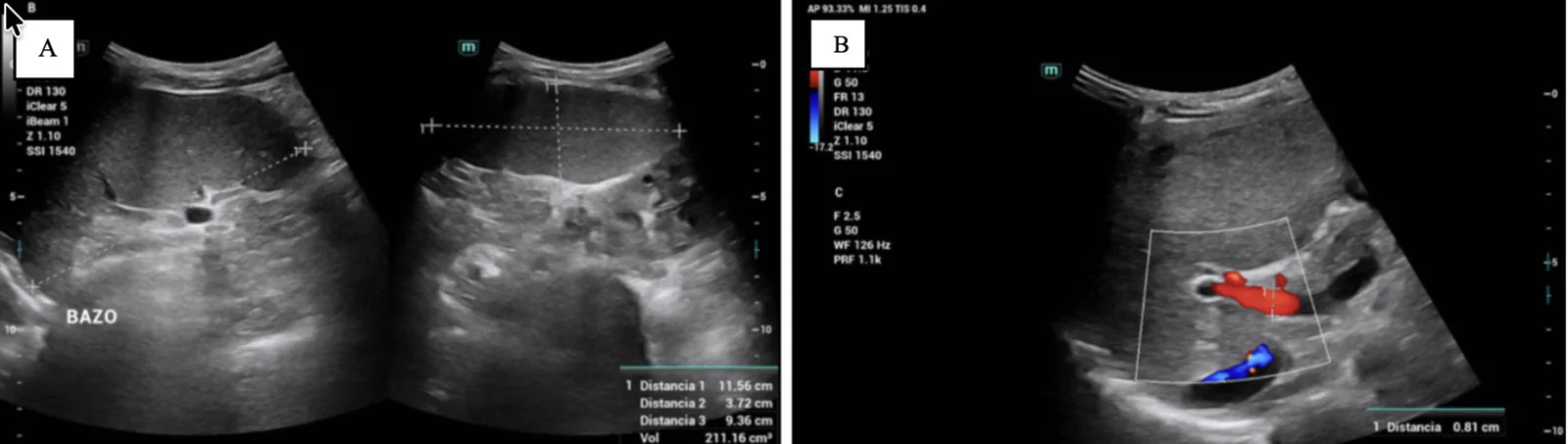
Imaging findings consistent with non-cirrhotic portal hypertension and splenomegaly. **(A)** abdominal imaging demonstrating splenomegaly, consistent with portal hypertension. The spleen shows preserved morphology with increased longitudinal diameter. **(B)** Doppler ultrasound evaluation of the portal circulation. Longitudinal ultrasound image of the spleen demonstrating splenomegaly. Color Doppler ultrasound showing hepatopetal flow within the portal venous system.

**Table 2:** Summary of imaging findings supporting the diagnosis of pediatric ADPKD with non-cirrhotic portal hypertension.

Imaging Modality	Key Findings	Diagnostic Significance
Portal Doppler ultrasound	Hepatosplenomegaly with heterogeneous hepatic echotexture; patent portal, splenic, and hepatic veins with hepatopetal flow; no portal vein thrombosis, portosystemic collaterals, or ascites; no hepatic cysts.	Demonstrates preserved portal venous patency and supports a presinusoidal form of non-cirrhotic portal hypertension.
Transient elastography (FibroScan®)	Liver stiffness: 6.4 kPa, consistent with mild fibrosis.	Demonstrates low liver stiffness despite clinically significant portal hypertension, supporting a presinusoidal mechanism.
Contrast-enhanced abdominal CT angiography	Patent spleno-porto-mesenteric venous axis without vascular thrombosis or portosystemic shunts; normal biliary tract; no hepatic cysts; multiple bilateral renal cortical cysts.	Excludes vascular causes of portal hypertension and confirms associated renal involvement compatible with ADPKD.
Renal ultrasound	Multiple bilateral simple cortical cysts (approximately 12 in the right kidney and 11–13 in the left kidney); no hydronephrosis.	Imaging findings consistent with ADPKD.
Abdominal CT scan	Preserved liver morphology without focal lesions or hepatic cysts; hepatosplenomegaly; patent portal venous system; multiple bilateral renal cortical cysts; no ascites or lymphadenopathy.	Supports non-cirrhotic portal hypertension associated with ADPKD while excluding alternative intra-abdominal pathology.
Follow-up portal Doppler ultrasound	Persistent splenomegaly with preserved hepatopetal portal flow and patent portal venous system.	Demonstrates stable vascular findings during follow-up.

Abdominal computed tomography and renal ultrasound demonstrated normal-sized kidneys containing approximately 25 bilateral cortical cysts, the largest measuring 27 mm. The combination of clinically significant non-cirrhotic portal hypertension, preserved liver synthetic function, mild portal fibrosis, and cystic kidney disease suggested an underlying hepatorenal ciliopathy.

Whole-exome sequencing performed using peripheral blood genomic DNA identified a heterozygous PKD1 variant (NM_001009944.3:c.4897G > T), resulting in the truncating variant p.Glu1633Ter. According to ACMG criteria, the variant was classified as pathogenic based on the PVS1, PM2, and PP4 criteria. No additional pathogenic variants or copy number alterations involving genes associated with hepatorenal ciliopathies were detected. Integration of the clinical, radiologic, histopathologic, and molecular findings established the diagnosis of autosomal dominant polycystic kidney disease with atypical hepatic involvement.

Because of the autosomal dominant inheritance pattern, both parents underwent screening renal ultrasonography, which showed no evidence of renal cysts. Parental genetic testing is currently underway to determine whether the identified PKD1 variant represents a *de novo* event or an inherited variant with incomplete penetrance or parental mosaicism.

At the most recent follow-up, at 10 years of age, the patient remained clinically stable and normotensive, with preserved liver and kidney function (Table 1). Since the initial diagnosis of portal hypertension, she has undergone three endoscopic variceal ligation procedures without recurrent gastrointestinal bleeding. She continues receiving propranolol (2 mg/kg/day divided every 12 h) as secondary prophylaxis and remains under regular endoscopic surveillance. After evaluation by a multidisciplinary liver transplant team, conservative management with close follow-up by pediatric hepatology and nephrology was recommended given her preserved hepatic function, stable clinical course, and absence of further bleeding episodes.

## Discussion

Autosomal dominant polycystic kidney disease (ADPKD) is a multisystemic ciliopathy caused by pathogenic variants in *PKD1 or PKD2*, which encode polycystin-1 (PC1) and polycystin-2 (PC2), respectively, two proteins that play a central role in primary cilium-mediated mechanosensation and intracellular signaling (1–3). Although ADPKD has traditionally been regarded as an adult-onset disease, accumulating evidence has demonstrated that its phenotypic spectrum extends into childhood and even the neonatal period, particularly in patients with very early-onset ADPKD (VEO-ADPKD) (1, 2, 11). The present case further expands this spectrum by demonstrating that clinically significant non-cirrhotic portal hypertension may represent the initial manifestation of pediatric ADPKD, even in the absence of hepatic cysts and despite only mild histologic fibrosis.

The molecular findings also provide important insights into the genotype–phenotype correlation. The identified *PKD1* variant (NM_001009944.3:c.4897G > T; p.Glu1633Ter) introduces a premature termination codon predicted to trigger nonsense-mediated mRNA decay (NMD), resulting in loss of functional polycystin-1 (12). Should any truncated protein escape NMD and be translated, it would lack the transmembrane domains and intracellular C-terminal tail, regions that are essential for interaction with polycystin-2 and activation of calcium-dependent signaling pathways involved in epithelial differentiation, tubular morphogenesis, and tissue homeostasis (3, 12). Previous studies have consistently demonstrated that truncating *PKD1* variants are associated with earlier disease onset, larger kidney volumes, more severe renal phenotypes, and faster disease progression than non-truncating variants (13, 14). These observations suggest that extensive loss of polycystin-1 function may also contribute to atypical extrarenal manifestations, including severe hepatobiliary involvement.

To our knowledge, the *PKD1 p.Glu1633Ter* variant has not previously been reported in association with early-onset clinically significant portal hypertension or congenital hepatic fibrosis (CHF)-like hepatic involvement. Therefore, this case further broadens the phenotypic spectrum associated with truncating *PKD1* variants and highlights the importance of correlating molecular findings with the clinical phenotype when evaluating atypical presentations of pediatric ADPKD.

Although hepatic involvement in ADPKD is most commonly characterized by polycystic liver disease, an increasing number of reports have described patients presenting with congenital hepatic fibrosis-like changes and clinically significant portal hypertension, particularly in association with truncating *PKD1* variants (6, 11, 15). To place our findings into clinical context, we conducted a focused review of the published literature and summarized all previously reported pediatric cases of ADPKD associated with congenital hepatic fibrosis, clinically significant portal hypertension, or other major hepatic manifestations (Table 3). Across these reports, the most common clinical findings included splenomegaly, hypersplenism, clinically significant portal hypertension, and preserved liver synthetic function, whereas hepatic cysts were absent or minimally expressed in several patients (6, 11, 15). However, our patient is unique in presenting with severe upper gastrointestinal bleeding secondary to grade III esophageal varices as the initial manifestation of disease in association with the truncating *PKD1* p.Glu1633Ter variant, mild histologic fibrosis (METAVIR F1), low liver stiffness (6.4 kPa), and complete absence of hepatic cysts, thereby further expanding the recognized hepatic phenotype of pediatric ADPKD.

**Table 3:** Published pediatric cases of ADPKD with congenital hepatic fibrosis or portal hypertension.

Study	Patient (s)	Age at presentation	Hepatic manifestations	Renal phenotype	Genetic findings	Main contribution
Lee & Paes, 1985 (19)	1 pediatric patient	Childhood	Congenital hepatic fibrosis with portal hypertension	Familial ADPKD	Not available	One of the earliest reports linking ADPKD with congenital hepatic fibrosis.
Cobben et al., 1990 (20)	Familial series	Childhood/adulthood	Congenital hepatic fibrosis and portal hypertension	Familial ADPKD	Not available	Demonstrated familial segregation and phenotypic variability.
Lipschitz et al., 1993 (21)	Pediatric patient	4 years	Early portal hypertension and congenital hepatic fibrosis	Initially minimal renal involvement	Not reported	Suggested that hepatic disease may precede overt renal manifestations.
O'Brien et al., 2012 (6)	19 patients from 14 families	Pediatric and adult	Splenomegaly, hypersplenism, variceal bleeding, congenital hepatic fibrosis	Variable renal disease	PKD1 variants identified in several families	Largest published cohort of ADPKD associated with congenital hepatic fibrosis.
Sila et al., 2023 (15)	8-year-old child	8 years	Congenital hepatic fibrosis presenting with portal hypertension	Familial ADPKD	Genetic evaluation reported	Recent pediatric case supporting early hepatic presentation.

Collectively, these observations reinforce the growing recognition that clinically significant hepatic involvement may occasionally represent an early manifestation of ADPKD, particularly in very early-onset disease (10, 11). Consequently, ADPKD should be considered in the differential diagnosis of children presenting with otherwise unexplained non-cirrhotic portal hypertension, particularly when renal cysts or other features suggestive of an underlying hepatorenal ciliopathy are identified.

A key feature of our patient was the presence of clinically significant portal hypertension despite preserved hepatocellular function, a pattern highly suggestive of a presinusoidal mechanism consistent with congenital hepatic fibrosis (CHF). Unlike the polycystic liver disease typically observed in adults with ADPKD, which is characterized predominantly by progressive cyst formation with relatively limited hemodynamic consequences, CHF results from a ductal plate malformation leading to portal fibrosis, abnormal remodeling of the intrahepatic bile ducts, and progressive portal hypertension while preserving hepatic synthetic function (7–9). Although CHF is classically associated with autosomal recessive polycystic kidney disease (ARPKD), increasing evidence indicates that a similar hepatic phenotype may also occur in selected patients with ADPKD, particularly those carrying truncating *PKD1* variants (6, 11, 15).

In our patient, liver biopsy demonstrated only mild portal fibrosis (METAVIR F1), whereas transient elastography revealed a liver stiffness of 6.4 kPa, also consistent with mild fibrosis. The discordance between clinically significant portal hypertension and relatively limited fibrosis is noteworthy and has been recognized in presinusoidal forms of non-cirrhotic portal hypertension. In these disorders, increased portal pressure primarily reflects abnormalities of the portal vascular architecture and intrahepatic vascular resistance rather than advanced diffuse fibrosis (7–9). Consequently, liver stiffness measurements may remain lower than expected despite clinically significant portal hypertension, emphasizing that transient elastography should always be interpreted within the broader clinical, radiologic, and hemodynamic context.

Similarly, liver biopsy has well-recognized limitations in the diagnosis of congenital hepatic fibrosis because ductal plate malformations and portal tract abnormalities may be heterogeneously distributed throughout the liver, making histopathologic assessment susceptible to sampling variability (8, 9). Therefore, histopathologic findings alone neither confirm nor exclude the diagnosis of CHF. Instead, diagnosis should be established through integration of the clinical presentation, evidence of portal hypertension, imaging findings, liver stiffness assessment, and compatible molecular results. In the present case, the combination of splenomegaly, grade III esophageal varices, preserved liver synthetic function, characteristic imaging findings, and identification of a pathogenic truncating *PKD1* variant strongly supports the diagnosis of congenital hepatic fibrosis-like hepatic involvement associated with ADPKD.

Although definitive hemodynamic characterization of presinusoidal portal hypertension is conventionally achieved by measurement of the hepatic venous pressure gradient (HVPG), this technique primarily reflects sinusoidal portal pressure and has limited diagnostic value in presinusoidal disorders such as congenital hepatic fibrosis and porto-sinusoidal vascular disease (16). Consequently, HVPG measurement would have been unlikely to modify either the diagnosis or the therapeutic management of our patient, whose diagnosis was established through the integration of clinical evidence of portal hypertension, imaging findings, liver histopathology, transient elastography, and molecular confirmation. Nevertheless, the absence of invasive hemodynamic assessment should be acknowledged as a limitation of this report.

From a pathophysiological perspective, defective polycystin signaling may contribute to abnormal remodeling of the ductal plate and portal mesenchyme during embryonic development. Altered cholangiocyte differentiation, impaired intracellular calcium signaling, and abnormal epithelial–mesenchymal interactions may promote portal tract remodeling and increased intrahepatic vascular resistance before extensive fibrosis develops (8, 17). These mechanisms provide a biologically plausible explanation for the development of clinically significant portal hypertension despite only mild histologic fibrosis.

The differential diagnosis of pediatric non-cirrhotic portal hypertension is broad and includes vascular disorders, congenital hepatic fibrosis associated with autosomal recessive polycystic kidney disease (ARPKD), porto-sinusoidal vascular disease, hematologic disorders, immunologic diseases, metabolic disorders, infectious conditions, and drug-induced liver injury. In our patient, these alternative etiologies were systematically excluded through clinical evaluation, laboratory testing, imaging studies, and comprehensive molecular analysis, supporting *PKD1*-associated ADPKD as the most likely underlying diagnosis. Importantly, the absence of hepatic cysts should not exclude clinically significant hepatic involvement in ADPKD, as reliance on cystic liver disease alone may delay recognition of these uncommon pediatric presentations (6, 10, 11).

From a clinical perspective, this case highlights the increasing role of molecular testing in the evaluation of children with unexplained non-cirrhotic portal hypertension. In patients presenting with portal hypertension accompanied by renal cysts or other features suggestive of a hepatorenal ciliopathy, early genetic testing for *PKD1 and PKD2* should be considered, as it may establish an etiologic diagnosis before the complete phenotypic spectrum becomes clinically apparent, facilitate multidisciplinary management, and enable appropriate long-term surveillance (1, 2, 13).

Our patient currently remains clinically stable, with preserved renal and hepatic function under multidisciplinary follow-up by pediatric nephrology and hepatology. Long-term surveillance remains essential because renal disease progression and hepatic complications may evolve independently over time (1, 2, 13). Although disease-modifying therapies such as tolvaptan have demonstrated benefit in selected adults with rapidly progressive ADPKD, experience in children remains limited, and treatment decisions should therefore be individualized (13). Likewise, continued multidisciplinary follow-up will be important to determine whether isolated kidney transplantation or combined liver–kidney transplantation may become appropriate should progressive renal disease or refractory portal hypertension develop.

This study has several limitations. As a single-case report, it cannot establish a definitive causal relationship between the identified PKD1 variant and the observed hepatic phenotype. Although liver biopsy was available, it has inherent limitations for evaluating the complete portal architecture and may underestimate congenital hepatic fibrosis because of sampling variability (8, 9). In addition, parental genetic testing is still pending, precluding definitive determination of whether the identified variant represents a *de novo* event, parental mosaicism, or inherited disease with incomplete penetrance. Although HVPG was not performed, its absence is unlikely to have altered either the diagnostic interpretation or the therapeutic management, given the clinical, radiologic, histopathologic, elastographic, and molecular evidence supporting a presinusoidal mechanism of portal hypertension (18). Nevertheless, these limitations should be considered when interpreting our findings.

Taken together, these findings reinforce the emerging recognition that pediatric ADPKD may present with atypical hepatic manifestations beyond classical polycystic liver disease. They also highlight the importance of integrating clinical, radiologic, histopathologic, and molecular findings when evaluating children with unexplained non-cirrhotic portal hypertension, particularly when features suggestive of an underlying hepatorenal ciliopathy are present.

## Conclusion

This case broadens the recognized clinical spectrum of pediatric autosomal dominant polycystic kidney disease by showing that a truncating PKD1 variant may present with clinically significant non-cirrhotic portal hypertension and variceal bleeding, even in the absence of hepatic cysts and despite only mild hepatic fibrosis. These findings emphasize that ADPKD should be considered in the differential diagnosis of children with otherwise unexplained portal hypertension when renal cysts or other features of a hepatorenal ciliopathy are present. Early molecular diagnosis may facilitate timely etiologic confirmation, guide multidisciplinary management, and further improve our understanding of genotype–phenotype correlations in pediatric ADPKD.

## Data Availability

The relevant clinical data supporting the conclusions of this case report are included in the article. The underlying patient records are not publicly available due to patient confidentiality and privacy restrictions. Further inquiries should be directed to the corresponding author.
